# Sunitinib-induced hypothyroidism predicts progression-free survival in metastatic renal cell carcinoma patients

**DOI:** 10.1007/s12032-017-0928-z

**Published:** 2017-03-25

**Authors:** Anna Buda-Nowak, Jakub Kucharz, Paulina Dumnicka, Marek Kuzniewski, Roman Maria Herman, Aneta L. Zygulska, Beata Kusnierz-Cabala

**Affiliations:** 1 Department of Oncology, University Hospital in Krakow, Sniadeckich 10, 31-531 Cracow, Poland; 2 Department of Experimental and Clinical Surgery, Jagiellonian University Medical College, Michalowskiego 12, Cracow, 31-126 Poland; 3 Department of Uro-Oncology, Maria Sklodowska-Curie Memorial Cancer Center and Institute of Oncology, Roentgena 5, 02-781 Warsaw, Poland; 4 Department of Medical Diagnostics, Jagiellonian University Medical College, Medyczna 9, 30-688 Cracow, Poland; 5 Department of Nephrology, Jagiellonian University Medical College, Kopernika 15, 31-501 Cracow, Poland; 6 Department of Diagnostics, Chair of Clinical Biochemistry, Jagiellonian University Medical College, Kopernika 15A, 31-501 Cracow, Poland

**Keywords:** Renal cell carcinoma, Hypothyroidism, Prognosis, Sunitinib, Predictive factor, Progression-free survival

## Abstract

Sunitinib is a tyrosine kinase inhibitor (TKI) used in treatment of metastatic renal cell carcinoma (mRCC), gastrointestinal stromal tumors and pancreatic neuroendocrine tumors. One of the most common side effects related to sunitinib is hypothyroidism. Recent trials suggest correlation between the incidence of hypothyroidism and treatment outcome in patients treated with TKI. This study evaluates whether development of hypothyroidism is a predictive marker of progression-free survival (PFS) in patients with mRCC treated with sunitinib. Twenty-seven patients diagnosed with clear cell mRCC, after nephrectomy and in ‘good’ or ‘intermediate’ MSKCC risk prognostic group, were included in the study. All patients received sunitinib as a first-line treatment on a standard schedule (initial dose 50 mg/day, 4 weeks on, 2 weeks off). The thyroid-stimulating hormone serum levels were obtained at the baseline and every 12 weeks of treatment. In statistic analyses, we used Kaplan–Meier method for assessment of progression-free survival; for comparison of survival, we used log-rank test. In our study, the incidence of hypothyroidism was 44%. The patients who had developed hypothyroidism had better median PFS to patients with normal thyroid function 28,3 months [95% (CI) 20.4–36.2 months] versus 9.8 months (6.4–13.1 months). In survival analysis, we perceive that thyroid dysfunction is a predictive factor of a progression-free survival (PFS). In the unified group of patients, the development of hypothyroidism during treatment with sunitinib is a positive marker for PFS. During that treatment, thyroid function should be evaluated regularly.

## Introduction

Sunitinib is an oral inhibitor of multiple tyrosine kinases of vascular endothelial growth factor receptors (VEGFR1, VEGFR2 and VEGFR3), platelet-derived growth factor receptors (PDGFRα and PDGFRβ), the stem cell factor KIT receptor, Fms-3-like tyrosine kinase (FLT3) receptor, the glial cell line-derived neurotrophic factor receptor (RET) and colony-stimulating factor 1 receptor (CSF-1R) [1–3].

Sunitinib was approved by the Food and Drug Administration (FDA) in treatment of advanced renal cell cancer (RCC), gastrointestinal stromal tumors (GISTs) and pancreatic neuroendocrine tumors [3]. In metastatic clear cell RCC, it is indicated by ESMO guidelines as a first-line treatment in patients with ‘good’ or ‘intermediate’ risk according to the Memorial Sloane Kettering Cancer Centre (MSKCC) and Heng criteria [4].

Treatment with the sunitinib is associated with favorable safety profile. However, as the treatment with other targeted agents, the side effects differ from the standard chemotherapy. The most common adverse events associated with the sunitinib treatment are cardiovascular toxicity (hypertension, decline in left ventricular ejection fraction), dermatologic toxicity (rash, hand-foot syndrome), fatigue and asthenia, gastrointestinal toxicity, myelosuppression and endocrine toxicity (hypothyroidism and adrenal insufficiency) [5, 6]. Although the severity of occurring side effects is mainly mild or moderate, they should be managed directly in order to prevent dose reduction or withdrawal of the treatment [6]. On the other hand, our recent study suggested correlation between the number of sunitinib-induced adverse effects and treatment outcome (PFS) [7]. Also the several trials confirmed that developing hypertension during treatment is associated with significant improvement in response rate, progression-free survival and overall survival [7–9]. Recent trials suggested correlation between the incidence of hypothyroidism and better outcome in patients treated with TKI [1, 2, 10]. The frequency of impaired thyroid function during treatment is estimated of 36–85% [11]. This study evaluates the effect of one of the most common side effects, hypothyroidism on efficacy of the treatment with sunitinib in mRCC.

## Materials and methods

The retrospective study included 27 patients treated at the Department of Oncology, University Hospital in Krakow. The inclusion criteria were as follows: diagnosis of stage IV clear cell mRCC, application of sunitinib as first-line treatment for the mRCC, prior nephrectomy (total or nephron sparing surgery) and good or intermediate Memorial Sloane Kettering Cancer Centre (MSKCC) risk prognosis (Table 1). All patients received sunitinib on a standard schedule (initial dose 50 mg/day, 4 weeks on, 2 weeks off). The thyroid-stimulating hormone (TSH) serum concentrations were obtained at the baseline and every 12 weeks (i.e., after every second cycle of treatment). TSH was measured on the day of blood collection with electrochemiluminescence immunoassay (ECLIA) on Cobas 600 analyzer (Roche Diagnostics). The reference interval for TSH was 0.27–4.20 µIU/mL. Hypothyroidism was defined as serum TSH > 4.20 µIU/mL. Patients with hypothyroidism were referred to endocrinologist, and appropriate treatment was initiated according to the current guidelines.

**Table 1:** Study group characteristics

Age (years)	65 (59–69)
Gender: women/man [n(%)]	9 (33)/18 (67)
Body mass (kg)	74 (67–84)
BMI (kg/m^2^)	27 (25–30)
Disseminated disease at baseline [*n* (%)]	22 (81)
Fuhrman grade >2 [*n* (%)]	17 (63)
Time from diagnosis to the systemic treatment <1 year [*n* (%)]	13 (48)
MSKCC prognosis: favorable/intermediate [*n* (%)]	21 (78)/6 (22)
IMRDC prognosis: favorable/intermediate [*n* (%)]	19 (70)/8 (30)
ECOG performance status (PS) before the treatment with sunitinib 0/1 [*n* (%)]	13 (48)/14 (52)
Positive history of hypertension [*n* (%)]	3 (11)
Positive history of hypothyroidism [*n* (%)]	0

We received permission to conduct this study from the Jagiellonian University Bioethics Committee (Permission No. KBET/45/B/2013). For this type of study, formal consent is not required.

Statistical analysis was performed using Statistica 12 software (StatSoft Inc., Tulsa, USA). Data were reported as number of patients (percentage of the group) for categories and median (lower–upper quartile) for quantitative variables. Progression-free survival was counted from the start of treatment with sunitinib until disease progression or death from any cause, or was censored at the end of the study. PFS was estimated with Kaplan–Meier method and compared with log-rank test. Also, Cox proportional hazard regression was used to assess independent predictors of PFS. The tests were two-tailed, and the results at *p* < 0.05 were interpreted as statistically significant.

## Results

**Fig. 1:**
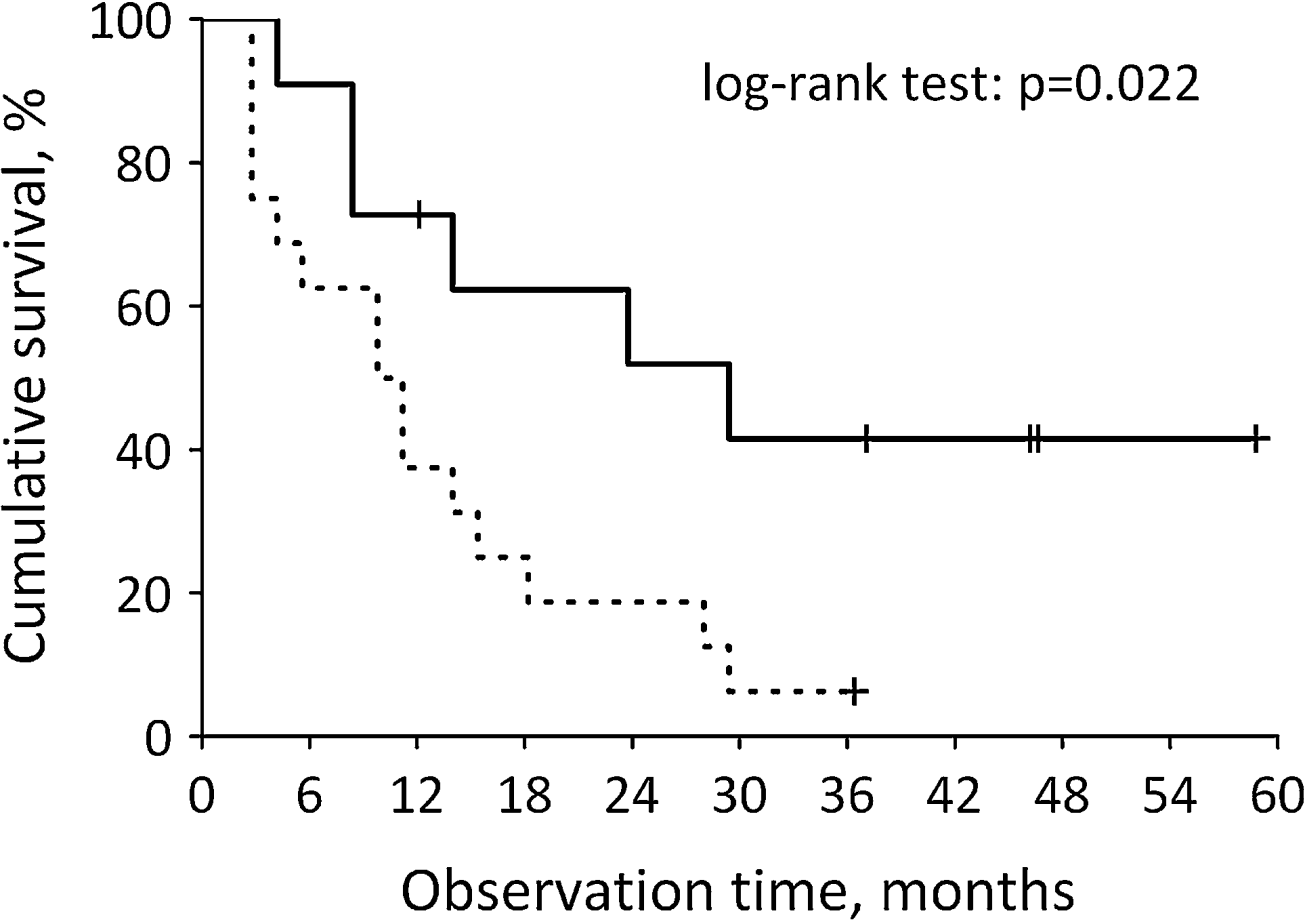
Cumulative survival in patients who developed hypothyroidism during sunitinib treatment (*solid line*) and those who did not (*dashed line*)

All of the patients included in the study were tested for thyroid function before starting sunitinib and after every second course of treatment. None of the patients had positive medical history for hypothyroidism. Baseline TSH was within reference interval in all patients: median TSH before starting sunitinib was 1.56 (1.08–2.00) µIU/mL. In the course of treatment, serum TSH increased above the upper reference limit in 12 patients (44%). Median TSH after second cycle of treatment was 4.90 (1.77–10.30) µIU/mL. The incidence of hypothyroidism during sunitinib treatment was associated with longer progression-free survival of 28,3 months [95% (CI) 20.4–36.2 months] versus 9.8 months (6.4–13.1 months) (*p* = 0.022) (Fig. 1). Moreover, the incidence of hypothyroidism predicted PFS independently of MSKCC score (hazard ratio: 0.37; 95% CI 0.14–0.98; *p* = 0.045).

## Discussion

The mechanism of thyroid dysfunction during TKI treatment has been yet not fully defined. The most probable mechanism could be connected with VEGFR blockade. The thyroid gland is well vascularized with VEGFR signaling-dependent blood flow that is crucial for thyroid function. Sunitinib by inhibiting the VEGFR receptor may influence on the vascularity of the gland and consequently induce tissue ischemia, which results in thyroid insufficiency [2, 12]. Other considered mechanisms are sunitinib-induced thyrotoxicosis, iodine uptake blocking, increased activity of deiodinase 3, inhibition of thyroperoxidase and increased metabolism of levothyroxine [1, 2, 13–18]. One of the recent studies analyzing hypothyroidism during treatment with sunitinib and sorafenib suggested a new hypothesis. Study revealed that sunitinib specifically binds to three retinoid receptors. Based on the fact that thyroid hormone action is mediated by thyroid hormone receptors and retinoid acid receptors, it is possible that sunitinib competes with thyroid hormone receptors for binding with retinoic acid receptors which finally results in hormonal dysregulation [19].

In our study, the incidence of hypothyroidism during sunitinib treatment was 44% (12 patients).

There was a statistically significant difference in median PFS between patients who developed hypothyroidism and patients without that side effect. That results suggest that hypothyroidism incidence during treatment with sunitinib was a predictive marker of a progression-free survival. The results are partly similar to the Wolter’s et al. who analyzed correlation between thyroid dysfunction and treatment outcome in a group of 40 patients enrolled in the study. Thyroid dysfunction was observed in 70% of patients, and 32.5% of them had symptomatic hypothyroidism. Statistical analysis revealed that thyroid dysfunction was associated with longer PFS and however was not correlated with OS [20].

Another study that supports our results is Kust et al.’s survey assessing influence of hypothyroidism on treatment response to sunitinib therapy. The incidence of hypothyroidism in their sample was 29.3%, while in our study—44%. The patients who developed hypothyroidism and receive substitution therapy had longer PFS compared to all other patients (25.3 vs. 9.0 months) [21]. Schmidinger et al.’s study which assessed clinical impact of hypothyroidism in patients with mRCC treated with sunitinib or sorafenib revealed correlation between hypothyroidism and percentage of overall response rate. The correlation was observed in both groups—treated with sunitinib and with sorafenib. In the group treated with sunitinib, there was no correlation between hypothyroidism and PFS nor OS. However, patients enrolled in the study got TKI in the second-line treatment which is the major difference between this and our study. Patients included in the study had progressed on cytokines and/or had received a TKI or monoclonal antibody before introducing sunitinib or sorafenib [10]. Also the TSH level was assessed after the first and second cycle, and according to recent studies, the time from beginning of the treatment with sunitinib to the onset of hypothyroidism is longer. According to Wong et al. [18], the average development of this side effect is 5 months. Vettel et al. have reported that the onset of hypothyroidism was observed between 12 and 50 weeks after initiation of sunitinib with growing incidence with treatment prolongation [11, 22]. Considering the relatively long time of hypothyroidism development, the early evaluation of treatment efficacy is limited. However, it may provide information about expected PFS in group of patients with occurrence of impaired thyroid function. In those cases, the supplementation of levothyroxine should be considered. Sunitinib dose reduction due to hypothyroidism should be avoided.

## Conclusion

Results of our study support previous assumptions about the correlation between incidence of hypothyroidism and treatment benefit. We found that in uniform group of patients (stage IV clear cell mRCC, after total or partial nephrectomy, ‘good’ or ‘intermediate’ MSKCC risk prognosis and sunitinib as a first-line treatment) the developing of hypothyroidism is a predictive marker of PFS. Consequently, the thyroid function should be evaluated regularly during treatment with sunitinib. Thyroid function check may eliminate the risk of assignment of the typical symptoms of hypothyroidism to cancer or to fatigue caused by sunitinib.

Early detection of hypothyroidism and beginning of levothyroxine substitution if necessary may avoid treatment interruptions or dose reductions.
